# A New Oxidative Stress Model, 2,2-Azobis(2-Amidinopropane) Dihydrochloride Induces Cardiovascular Damages in Chicken Embryo

**DOI:** 10.1371/journal.pone.0057732

**Published:** 2013-03-01

**Authors:** Rong-Rong He, Yan Li, Xiao-Di Li, Ruo-Nan Yi, Xiao-Yu Wang, Bun Tsoi, Kenneth Ka Ho Lee, Keiichi Abe, Xuesong Yang, Hiroshi Kurihara

**Affiliations:** 1 Pharmacy College, Jinan University, Guangzhou, People’s Republic of China; 2 Division of Histology and Embryology, Key Laboratory for Regenerative Medicine of the Ministry of Education, Medical College, Jinan University, People’s Republic of China; 3 Stem Cell and Regeneration Thematic Research Programme, School of Biomedical Sciences, Chinese University of Hong Kong, Hong Kong Special Administrative Region, China; 4 BRAND’s Health Science Centre, Scientific Research Division, Singapore, Singapore; Heart Science Centre, Imperial College London, United Kingdom

## Abstract

It is now well established that the developing embryo is very sensitive to oxidative stress, which is a contributing factor to pregnancy-related disorders. However, little is known about the effects of reactive oxygen species (ROS) on the embryonic cardiovascular system due to a lack of appropriate ROS control method in the placenta. In this study, a small molecule called 2,2-azobis(2-amidinopropane) dihydrochloride (AAPH), a free radicals generator, was used to study the effects of oxidative stress on the cardiovascular system during chick embryo development. When nine-day-old (stage HH 35) chick embryos were treated with different concentrations of AAPH inside the air chamber, it was established that the LD_50_ value for AAPH was 10 µmol/egg. At this concentration, AAPH was found to significantly reduce the density of blood vessel plexus that was developed in the chorioallantoic membrane (CAM) of HH 35 chick embryos. Impacts of AAPH on younger embryos were also examined and discovered that it inhibited the development of vascular plexus on yolk sac in HH 18 embryos. AAPH also dramatically repressed the development of blood islands in HH 3+ embryos. These results implied that AAPH-induced oxidative stress could impair the whole developmental processes associated with vasculogenesis and angiogenesis. Furthermore, we observed heart enlargement in the HH 40 embryo following AAPH treatment, where the left ventricle and interventricular septum were found to be thickened in a dose-dependent manner due to myocardiac cell hypertrophy. In conclusion, oxidative stress, induced by AAPH, could lead to damage of the cardiovascular system in the developing chick embryo. The current study also provided a new developmental model, as an alternative for animal and cell models, for testing small molecules and drugs that have anti-oxidative activities.

## Introduction

Oxidative stress is regarded as a consequence of an imbalance between the production of reactive oxygen species (ROS) and antioxidant defense. It is an important mediator of cellular and structural damages that include the membrane, proteins, lipids and DNA [1]. During pregnancy, the occurrence of oxidative stress was favored by the richly developed vascularization and abundant mitochondria in the placenta [2]. However, the accumulation ROS in the developing embryo could also cause increased cell apoptosis and alter gene expressions [3], which may induce embryonic death and malformations, such as fetal intrauterine growth retardation [4]. Therefore, excess ROS has been proposed to cause severe damage during embryo development [5], especially in the cardiovascular system because it is one of the first functional systems to be developed. However, there was no suitable model to evaluate the destructive effects of oxidative damage on the developing cardiovascular system.

The chick embryo can serve as an excellent *in vivo* model for investigating oxidative stress because of its low cost, similarity to mammalian embryos and easy accessibility. In addition, it also allows various parameters to be evaluated using molecular biology, cellular biology and surgical micro-manipulation [6]. Currently, there are several popular assays being used to study anti-oxidative stress, such as oxygen radical absorbance capacity (ORAC), trolox equivalent antioxidant capacity assay (TEAC), total oxyradical scavenging capacity assay (TOSC), peroxyl radical scavenging capacity assay (PSC), and ferric reducing antioxidant potential assay (FRAP). However, these methods only measure chemical reactions and cannot be extrapolated to represent activity *in vivo*. These methods also cannot provide information on the bioavailability, stability, tissue retention and reactivity of reagents and compounds under physiological conditions. In this context, we propose the chick embryo as a new model for studying oxidative stress which will permit a better evaluation of the potential protective effects of antioxidants.

To establish the oxidative stress model, it is important to select an appropriate source of free radicals. An extensively reported generator of free radicals, 2,2′-azobis(2-amidinopropane) dihydrochloride (AAPH), was used in this study. AAPH is a water-soluble azo small molecule that is often employed in the study of lipid peroxidation and for the characterization of antioxidants. Decomposition of AAPH produces one mole of nitrogen and two moles of carbon radicals. The carbon radicals could either combine to produce stable products or react with molecular oxygen to generate peroxyl radicals - maintaining a constant rate of free radical production in solution [7]. This small molecule has been used extensively, as a source of thermal free radical, in the study of oxidations of red blood cells, plasma, whole blood, HeLa cells, various tissues, and even the whole body [8]. It has been reported that AAPH can cause various types of pathological changes through cellular oxidative damage. Although chick embryos have been used in studying the teratogenicity of exposure to various stressors, such as butenolide, ethanol, tetrachlorodibenzo-p-dioxin (TCDD) etc. [9], there have to date no reports on the effect of oxidative stress induced by AAPH. Therefore, the aim of this study is to examine the potential adverse effects of AAPH on embryonic cardiovascular system during development, in addition, to evaluate the efficacy of using the chick embryo as a new model for studying oxidative stress.

## Results

### Toxicity of AAPH on Chick Embryos

**Figure 1:**
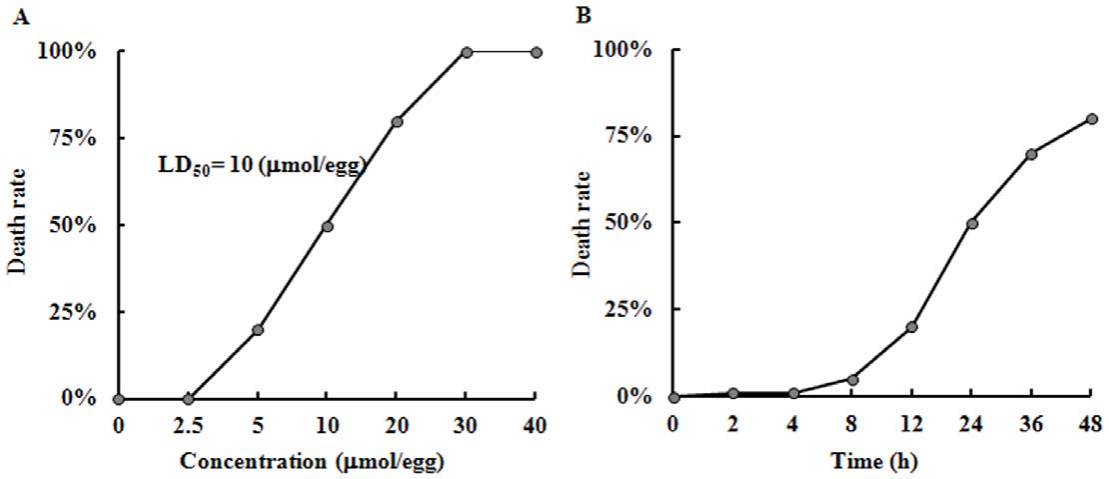
Embryo mortality rate in presence of different AAPH concentration. (A) AAPH (0–40 µmol/egg) were injected into the air chamber of eggs containing 9-day-old (stage HH 35) chick embryos and incubated for 24 hours. (B) Stage HH 35 chick embryos were treated with AAPH (10 µmol/egg) and cultured for 2–48 hours. The experiments were performed in triplicates with 20 eggs assigned for each group. Embryos without heart beat were deemed dead.

Serial dilution of water-soluble AAPH (40, 30, 20, 10, 5, 2.5 µmol/egg) were injected into the air chamber of 9-day-old (HH 35) chick embryos and incubated for 24 h. In the control, the eggs were injected with simple saline. Embryos were deemed dead if they did not exhibit heartbeat. The mortality rate of AAPH treated embryos appeared to increase in a dose dependent manner, as shown in Figure 1A. The LD_50_ of AAPH treatment was calculated as 10 µmol/egg. The mortality rate of embryos exposed to LD_50_ of AAPH for 2, 4, 8, 12, 24, 36 and 48 hours was presented in Figure 1B. It was found that the mortality rate rose dramatically when AAPH treatment time was extended from 12 hours onwards.

### Effects of AAPH on the Vasculature of Chorioallantoic Membrane (CAM) in Chick Embryos

**Figure 2:**
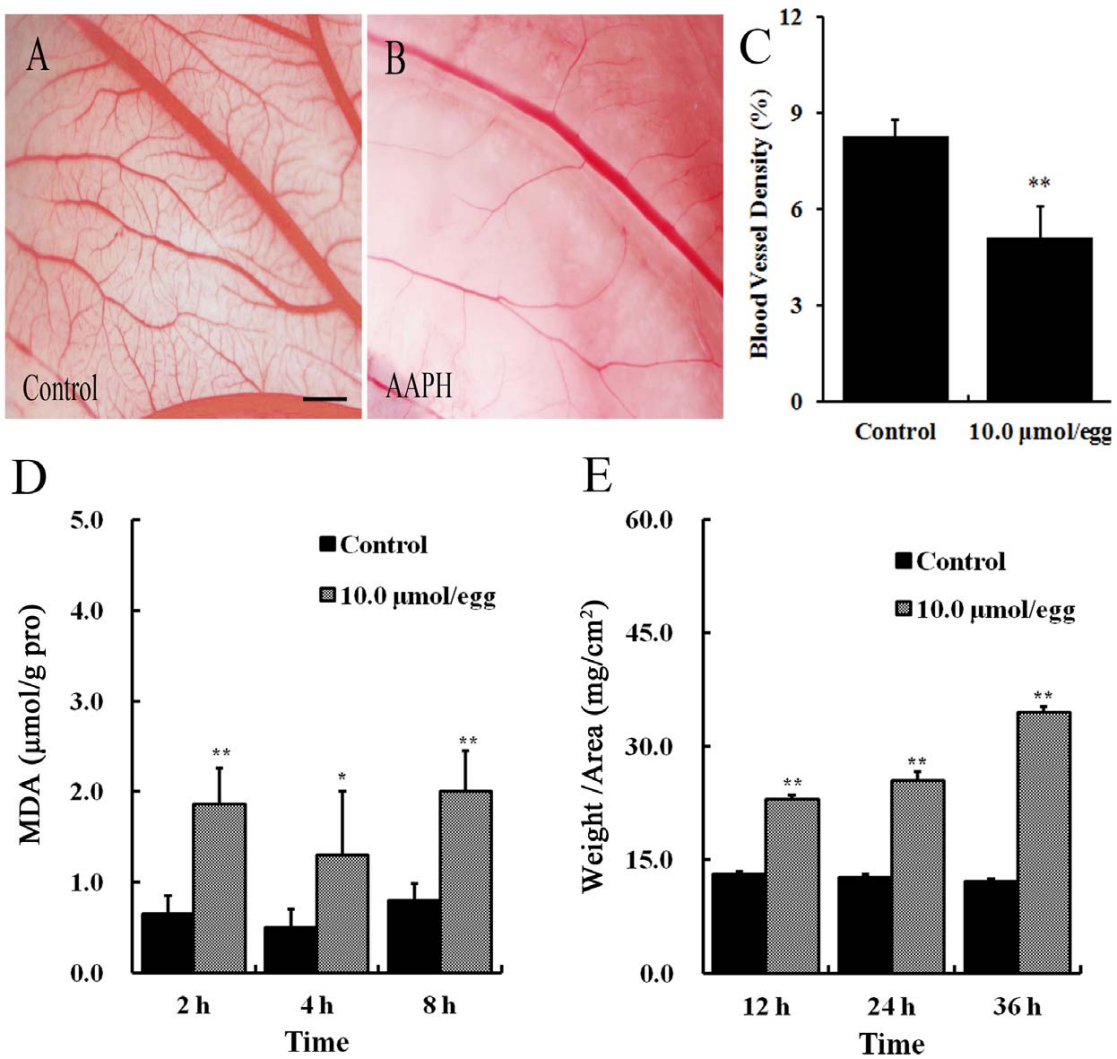
Effect of AAPH on angiogenesis in the CAM of chick embryos. (A) Representative appearance of CAM treated with saline for 24 h. (B) Representative appearance of CAM treated with 10 µmol/egg of AAPH for 24 h. (C) Statistical chart showing the blood vessel density of CAM from AAPH treated and untreated embryos. (D) The CAMs of embryos were treated with 10 µmol/egg of AAPH for 2–8 hours and then the MDA content of embryos were measured. (E) The thickness of CAM after 12–36 h exposure to AAPH. CAM thickness was calculated as the ratio of weight to area of CAM (mg/cm^2^). The results are presented as mean ± S.D (n = 10). Statistical significances were evaluated using SPSS13.5 software, presented as *p<0.05, **p<0.01 in comparison with control group. Scale bar = 1 mm.

Chick embryos (HH 35) were treated with 10 µmol of AAPH solution at volume of 100 µL for 24 h and then the CAMs were photographed (Figures 2A and B). It was found that AAPH treatment lead to a significant reduction in the density of CAM blood vessel plexus produced when compared with control (Figure 2C). Even a shortened exposure time (2 to 8 hours) of AAPH (10 µmol) on the embryos could still significantly elevate malondialdehyde (MDA) contents in CAM (Figure 2D). It was also determined that the thickness of CAM was significantly increased after being exposed to AAPH for more than 12 hours (Figure 2E). The results showed that AAPH caused oxidative damage to the blood vessel plexus in CAM of chick embryos. It implied that AAPH might have affected angiogenesis and vasculogenesis during the growth and development of chick embryos.

### Effects of AAPH on Yolk-sac Blood Vessels in Chick Embryos

**Figure 3:**
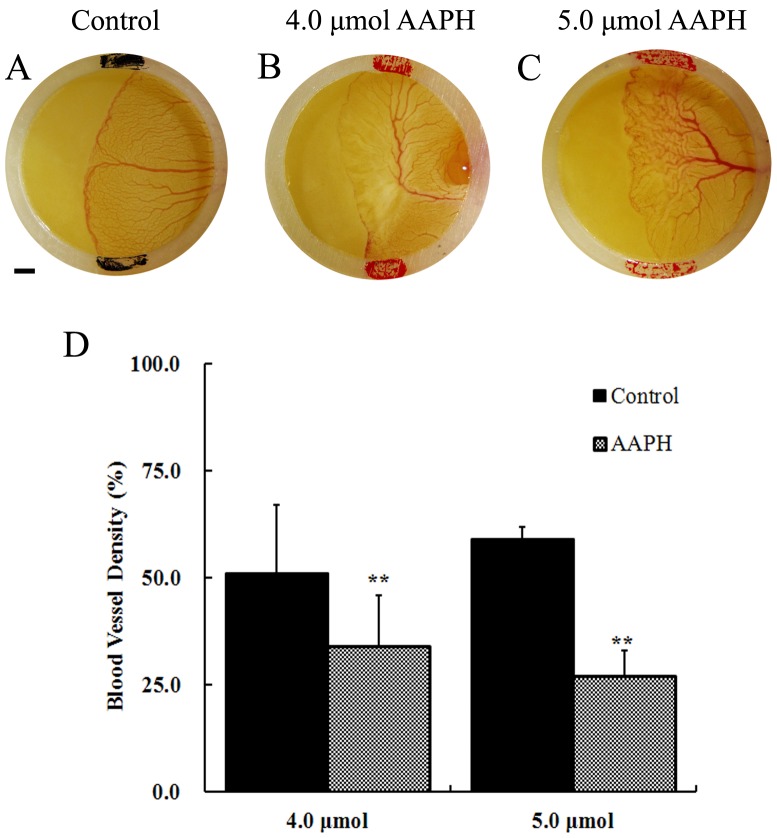
Effects of AAPH on yolk-sac blood vessels of chick embryo. (A) Representative appearance of yolk-sac blood vessels of HH 18 embryo treated with saline, (B) 4 µmol AAPH and (C) 5 µmol AAPH for 12 hours. The silastic rings in A–C had inside diameter at 9 mm and outside diameter at 11 mm. The yolk-sac blood vessel images were taken by a stereomicroscope (Olympus MVX10 with OPTPRO 2007 image acquisition system) with resolution ratio at 1024×768 (Scale bar = 1 mm). (D) Statistical chart showing the yolk-sac blood vessel density from AAPH treated and untreated embryos. The results represent the mean ± S.D (n = 10). Statistical significances were determined using SPSS13.5 software, **p<0.01 compared with control group.

The effect of AAPH-induced oxidation was also investigated in earlier stage chick embryos. Stage HH 18 chick embryos were mounted with silastic rings and treated with 4 or 5 µmol of AAPH for 12 hours. The effects of AAPH on yolk-sac blood vessel were then examined. The results revealed that AAPH exposure significantly inhibited the development of newly formed vascular plexus (Figures 3B and C) compared with the control (Figure 3A). There were fewer vessels (Figure 3B) and shrinkages in the leading edge of the vascular plexus (Figure 3C). The yolk-sac blood vessel density was also significantly decreased after AAPH treatment (Figure 3D). These results implied that AAPH inhibits angiogenesis in yolk sac of chick embryo.

### Effect of AAPH on Blood Islands Formation in Early Chick Embryo

**Figure 4:**
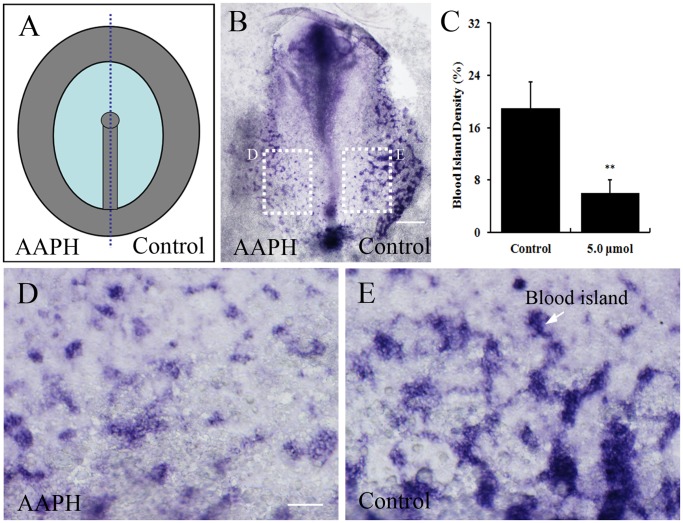
Effect of AAPH on blood islands formation in early chick embryos. (A) Schematic illustration of primitive streak stage chick embryos treated with AAPH (left side of embryo) and saline (right side of embryo). (B) *In situ* hybridization of whole-mount chick embryo revealing VE-Cadherin expression and extent of blood island formation. (C) Statistical chart showing the blood islands density of AAPH (5.0 µmol) treated and untreated sides of embryos. Results presented as mean ± S.D (n = 10). Data analyzed using SPSS13.5 software, **p<0.01 compared with control. (D) Magnified appearance of blood islands (white arrow) of left side of embryos treated with AAPH and (E) right side with saline. Scale bars = 1 mm in B and 100 µm in D–E.

The impact of AAPH on vasculogenesis was further investigated by observing blood islands formation in early stage of embryos. To evaluate the extent of blood islands formation, *in situ* hybridization was performed for vascular endothelial cadherin (VE-Cadherin) as the markers for blood islands in area opaca of chick embryo. The blood islands are derived from the mesodermal progenitor cells in the posterior primitive streak during gastrulation. Therefore, AAPH (5.0 µmol) was applied to half side of HH 3^+^ chick embryos during early chicken (EC) culture [10] as schematically illustrated in Figures 4A. The other side treated with simple saline was served as the control. After AAPH exposure for 24 hours, the extra-embryonic areas reached stage HH 8, where blood islands were mainly located. It was determined that AAPH exposure led to fewer blood islands being formed (Figures. 4B, C and D) in comparison to the saline-treated control (Figures 4B, C and E). These findings suggest that AAPH-induced oxidative stress not only has negative impact on angiogenesis, but also inhibits the process of blood islands formation (i.e. initiation of angiogenesis).

### Effects of AAPH Exposure on Heart Development

**Figure 5:**
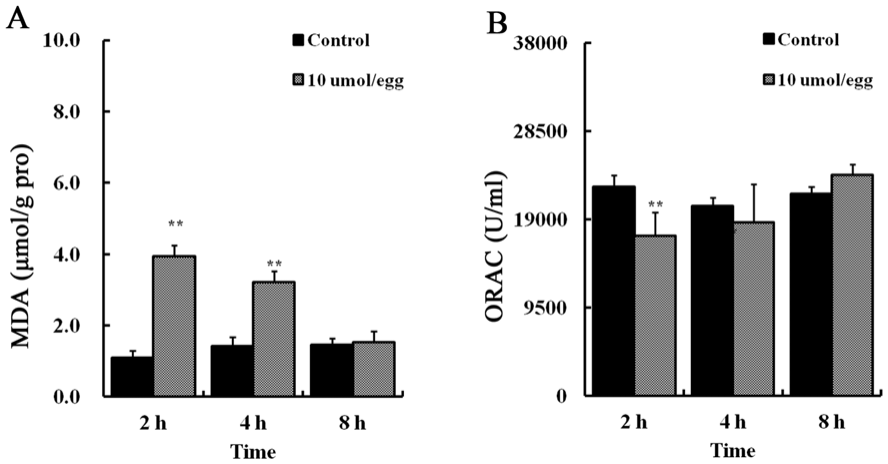
AAPH induces oxidative stress in the heart of chick embryo. (A) Statistical charts of MDA and (B) ORAC level in the heart of embryos treated with AAPH (10 µmol/egg) and saline. Results represent mean ± S.D (n = 10). Analyzed by SPSS13.5 software, **p<0.01 compared with control.

**Figure 6:**
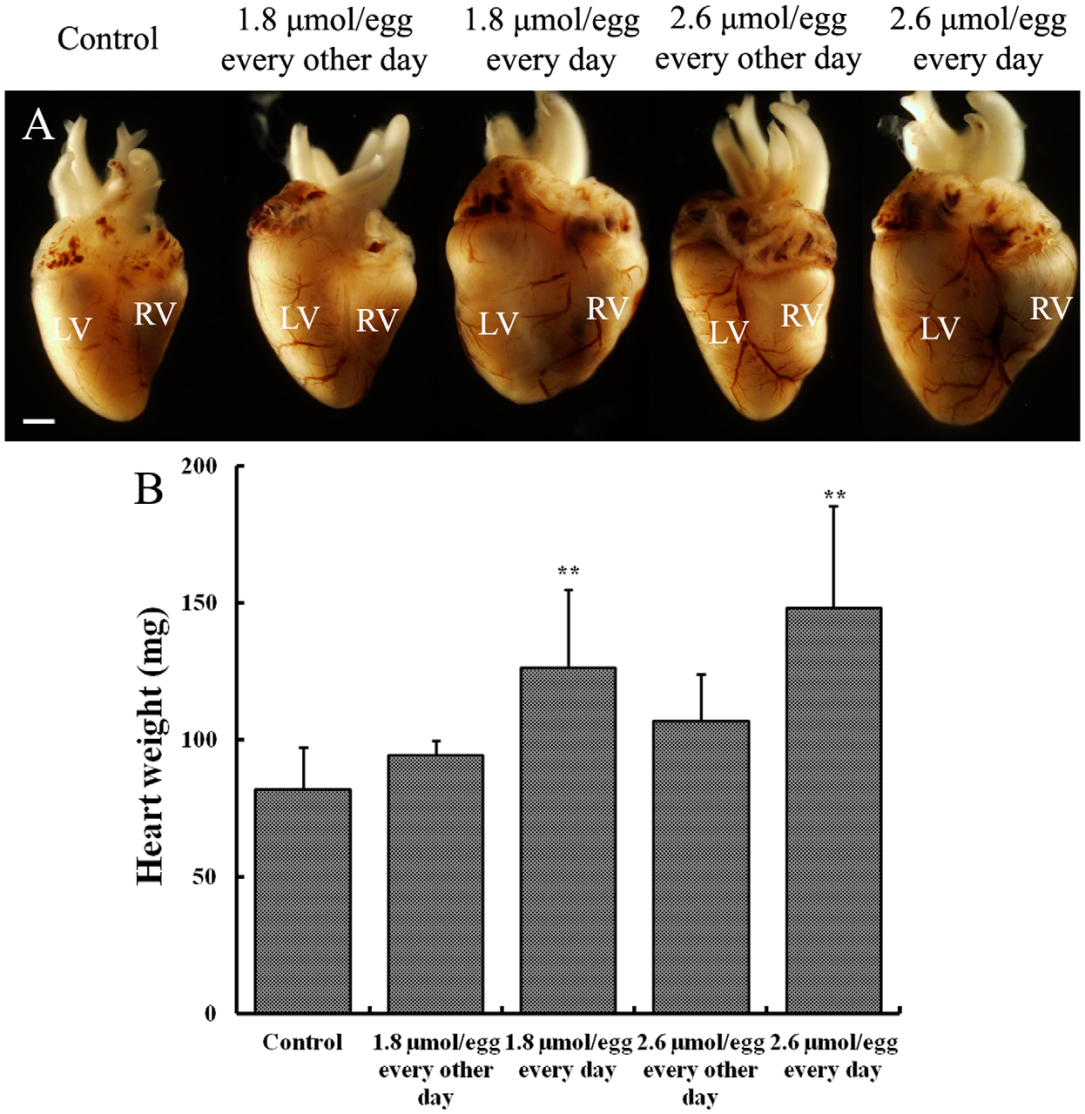
Effect of AAPH exposure on heart size and weight of chick embryos. (A) Representative appearance of hearts harvested from 14-day-old (HH 40) embryos treated with saline, 1.8 and 2.6 µmol of AAPH. (B) Statistical chart showing the embryonic heart weight after AAPH exposure. Results presented as mean ± S.D. (n = 10). Analyzed by SPSS13.5 software, **p<0.01 compared with control. Abbreviation: LV, left ventricle; RV, right ventricle. Scale bar = 1 mm.

**Figure 7:**
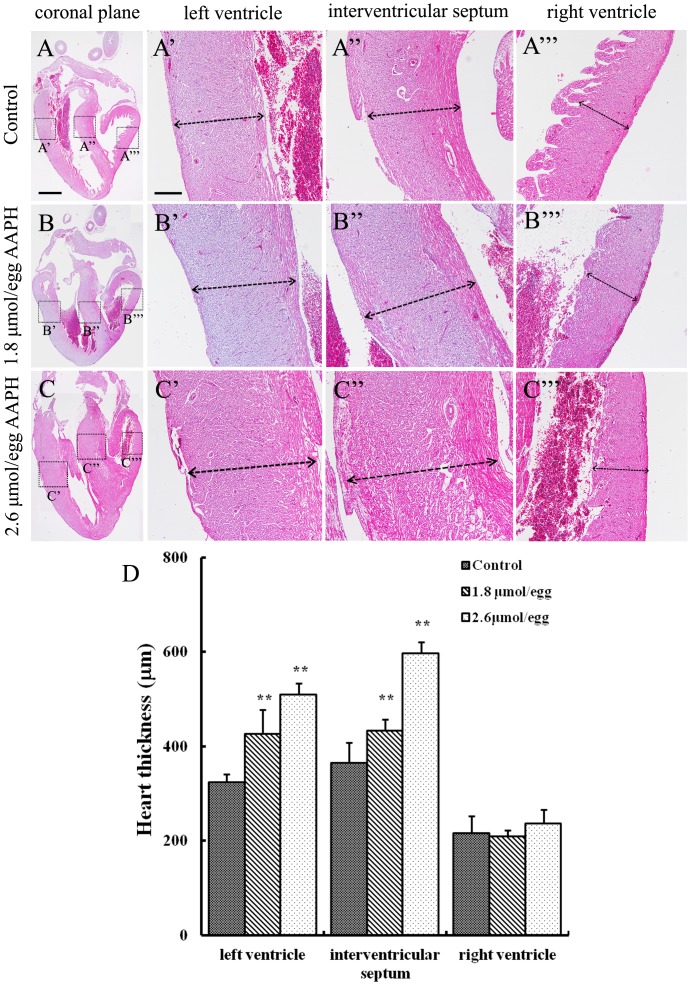
Effect of AAPH exposure on wall thickness of chick embryonic heart. (A–C) Haematoxylin- and eosin-stained histological vertical sections of embryonic hearts treated with (A) saline, (B) 1.8 µmol AAPH and (C) 2.6 µmol AAPH over 5 days. All histologic sections are presented with the atria on top and the left ventricle on the left. High magnification of (A’, B’ and C’) left ventricle, (A’’, B’’ and C’’) interventricular septum, and (A’’’, B’’’ and C’’’) right ventricle. (D) Statistical chart showing the thickness of the left and right ventricular walls and thickness of the interventricular septum. Results presented as mean ± S.D (n = 10). Data analyzed using SPSS13.5 software, **p<0.01 compared with control. Scale bars = 1mm in A–C and 500 µm in A’–A’’’, B’-B’’’ and C’–C’’’.

**Figure 8:**
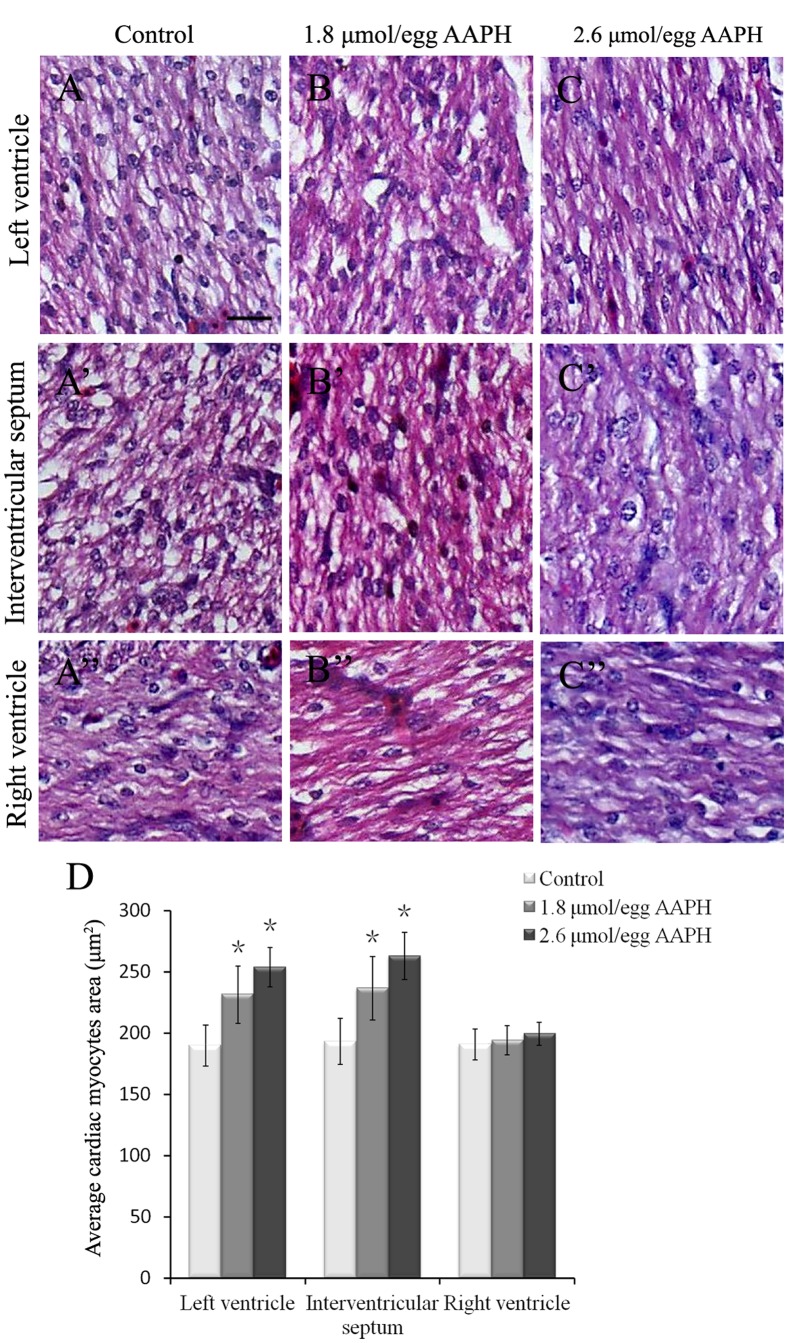
Effect of AAPH on cardiac myocytes size of chick embryo. (A–C) H&E-staining of heart sections obtained from left ventricle of HH 40 stage chick embryo of (A) control, (B) 1.8 µmol/egg AAPH and (C) 2.6 µmol/egg AAPH group. (A’–C’) H&E-staining of interventricular septum from HH 40 stage chick embryo of (A’) control, (B’) 1.8 µmol/egg AAPH and (C’) 2.6 µmol/egg AAPH group. (A’’–C’’) H&E-staining of right ventricle from HH 40 stage chick embryo of (A’’) control, (B’’) 1.8 µmol/egg AAPH and (C’’) 2.6 µmol/egg AAPH group. (D) Statistical data of average cardiac myocytes surface area. Results presented as mean ± S.D (n = 10) calculated by SPSS13.5 software, *p<0.05. Scale bar = 20 µm in A–C, A’–C’, A’’–C’’.

It was determined that AAPH (10 µmol/egg) exposure for 2 hours significantly elevated MDA content in the HH35 embryonic heart (Figure 5A) and ORAC level was significantly decreased (Figure 5B). Interestingly, ORAC level (antioxidant indicator) was recovered after 8 hour AAPH treatment. In addition, the size of the developing heart was found larger following lower doses of AAPH exposure. This was especially obvious when the HH 35 embryos were treated with 1.8 or 2.6 µmol/egg AAPH for 5 consecutive days (Figure 6A). However, exposing AAPH on alternative days at the same concentrations did not significantly affect the heart as compared with controls (Figure 6A). Consistent with the increase in heart size, there was a corresponding increase in heart weight (Figure 6B). However, it happened only if AAPH was given every day rather than on alternative days (i.e. AAPH exposure continually maintained at high levels). Haematoxylin-eosin (H&E) stained histological vertical sections of embryonic hearts were obtained from hearts shown in Figure 6A. We measured the thickness of the left and right ventricular walls and interventricular septum of AAPH (1.8 or 2.6 µmol/egg) treated heart at HH 40 (Figure 7). It was found that the left ventricular wall and interventricular septum were significantly thicker (Figures. 7B’–B’’ and C’–C’’) than the control (Figures. 7A’–A’’) in a dose-dependent manner (Figure 7D). This explained the increased heart size and weight following AAPH treatment (Figure 6). Interestingly, the wall thickness of right ventricle was not altered by AAPH treatment (Figures. 7A’’’, B’’’, C’’’ and D). Further experiments had performed on analysis of myocytes size, and results found that average myocytes size is increased in left ventricle and interventricular septum of both AAPH treatment groups (Figure 8 A–C, A’–C’). However, cell size did not vary in right ventricle between control and AAPH groups (Figure 8 A’’–C’’). Therefore, heart enlargement caused by AAPH was probably due to myocardic cell hypertrophy.

## Discussion

Oxidative stress is commonly considered as a causative factor of vascular injury and inflammation in many cardiovascular diseases, including hypertension and hyperlipidemia [11]. It has been demonstrated that excessive ROS production is involved in the etiology of vascular diseases [12], [13]. It was reported that a number of obstetric conditions are associated with deficient vasculogenesis in the developing placenta [14]. However, little is known of the effects of ROS on the developing cardiovascular system due to the lack of appropriate controls for ROS control method in the placenta. In our study, we have used AAPH to generate free radicals to elucidate the effects of oxidative stress on the developing cardiovascular system in chick embryos. This small molecule is a water soluble peroxyl radical generator and lipid per-oxidation initiator [15]. It can also produce carbon-centered radicals under aerobic conditions [16], [17]. Accordingly, we have used AAPH to produce free radicals to study the effects of oxidative stress on the developing cardiovascular system. From the data we obtained, it was established that the LD_50_ of AAPH exposure in stage HH 35 chick embryos was 10 µmol/egg.

The circulatory system is one of the first functional systems to develop. In the chick embryo, 2 sets of circulation system are formed during vascularization, including the CAM vascular circulation system and the yolk sac circulation system, which carry oxygen and nutrients to the embryos to maintain their growth. In this study, we have demonstrated that a brief exposure of AAPH significantly increased lipid peroxidation and inversely reduced blood vessel density in the CAM of chick embryos. Apart from CAM, the density of blood vessels on the yolk sac was also significantly reduced following AAPH exposure. The yolk-sac blood vessel system plays an important role in the transport of nutrients from the yolk to the embryo. These results implied that oxidative stress induced by AAPH was detrimental to angiogenesis in both CAM and yolk sac. Furthermore, AAPH exposure also disrupted vasculogenesis by repressing the formation of blood islands. Blood islands are developed from undifferentiated mesodermal cells that migrate into the area opaca of the yolk sac. These mesodermal cells differentiate into highly proliferative angioblasts that form solid angioblastic cords. The angioblastic cords then mature into endothelial cells, blood plasma and primitive erythroblasts. The outer cellular layer of the cords differentiates into endothelial cells while the blood plasma is derived via liquefaction of the central core during vessel lumen formation. The primitive erythroblasts emerge from the remnant cord masses or directly from the budding vascular endothelial cells. Therefore, factors (like free radicals generated by AAPH) that affect angiogenesis and blood island formation will have a major follow-on impact in cardiovascular development. Likewise, these free radicals could also diffuse into the developing heart via the blood vessels in the CAM and yolk sac to induce oxidative damages, which might also impact on the development of the heart in chick embryos. The above experimental results implied that AAPH-induced oxidative stress could impair the whole developmental processes associated with vasculogenesis and angiogenesis.

The heart is regarded as the first functional organ in the developing chick embryo. The development of the heart involves a multitude of morphogenetic mechanisms that includes cellular migrations, fusions, and tissue differentiation [18]. There are several methods that could be used to determine the oxidation level within tissues and cells. Measurements of ORAC level can reveal the anti-oxidation capacity of low molecular weight water-soluble antioxidants. Measurements of MDA (final metabolite of lipid peroxidation) content can indicate the extent of oxidative stress. In this study, we found a significant decrease in ORAC level and a significant increased MDA contents in the heart of embryos after only 2 h of AAPH treatment. However, the antioxidant system recovered after 8 hours of AAPH exposure, as the ORAC recovered back to normal levels. Nevertheless, this was sufficient to induce heart enlargement, where left ventricular walls and interventricular septum were thickened in a dose-dependent manner. Furthermore, myocytes size determination demonstrated that AAPH caused an enlarged cell surface area in left ventricle and interventricular septum, indicating the possibility of myocardiac cell hypertrophy. To date, there are numerous reports showing ROS can directly affect normal development because redox status can regulate key transcription factors and signaling pathways involved in cell proliferation, differentiation and apoptosis [19]. In this context, our results have shown that AAPH-induced oxidative stress could interfere with the crucial steps associated with chick heart morphogenesis which lead to myocardiac cell hypertrophy. Moreover, heart hypertrophy is often related with vascular abnormalities [20]. In our study, results have shown that AAPH-induced oxidative stress interfered blood islands formation, CAM and yolk sac vascular development. Therefore, AAPH not only could directly induce oxidation in the heart, but also indirectly influence the functions of embryonic heart through affecting vasculogenesis and angiogenesis during early stage of chick development. Altered vasculogenesis and angiogenesis could lead to vascular resistance, which formed a vicious cycle and might ultimately result in heart hypertrophy.

Our results have also produced a model which allows oxidative stress and damages to be studied in the developing embryonic cardiovascular system. It will also be useful to study the mechanisms of diseases induced by oxidative stress using this model. The chick embryo is an excellent *in vivo* model due to its low cost, similarity to mammalian embryos and is easily accessible. The chick embryo also has the advantage that it allows surgical micro-manipulation, genes can be over-expressed or silenced and cell migration to be tracked [21]. Because of all these advantages, the chick embryos have been used to assess numerous various harmful chemical substances [5]. Many chemical methods (such as ORAC) are now widely used for evaluating the antioxidative potential of natural products and food supplements. Although these chemical approaches are useful for high throughput screening, they do not demonstrate the metabolism and bioavailability of antioxidants, since they are not screened under physiological conditions. Biological systems are much more complex than simple chemical reactions in a test tube, and the effects of antioxidants may involve multiple targets and mechanisms. Therefore, it would be better using animal models for antioxidant assessment. Since various studies have presented large amount of information on organs and tissue development in the chick embryo and the genes involved, the chick embryo is an excellent model for elucidating the harmful effects of ROS and antioxidative potential of antioxidants to protect various organ systems from oxidative damage.

## Materials and Methods

### Chemicals and Reagents

2,2-azobis(2-amidinopropane) dihydrochloride (AAPH), sodium ﬂuorescein (FL) and 6-hydroxy-2,5,7,8-tetramethylchroman-2-carboxylic acid (trolox, a water soluble α-tocopherol analogue, served as a control standard) were purchased from Wako Pure Chemical Industries Ltd. (Osaka, Japan. Both Trolox and AAPH were dissolved in potassium phosphate buffer immediately before the ORAC assay. Coomassie brilliant blue kit and malondialdehyde (MDA) kit were purchased from Jiancheng Bioengineering Institute (Nanjing, China). NBT, BCIP, t-RNA, Boehringer blocking reagents, Anti-Digoxigenin-AP Fab fragments, DIG RNA Labeling Kit (SP6/T7) were purchased form Roche. EDTA, CHAPS, Heparin, Tween 20, Formamide, Deionised formamide, Eosin and Haematoxylin were purchased form Sigma (St. Louis, USA).

### AAPH Treatments of Chick Embryos

Fertilized leghorn eggs were acquired from the Avian Farm of South China Agriculture University (Guangzhou, China). The fertilized eggs were incubated in a humidified incubator (Yiheng Instruments, Shanghai, China) set at 38°C and 70% humidity, until the chick embryos research the desired Hamburger-Hamilton (HH) stage of development [22], [23]. Before experiment, the air chamber of eggs was marked and live embryos were selected under an egg candler. The shell above the air chamber was punched in a biosafety cabinet [24] and then different concentrations of AAPH (0, 2.5, 5, 10, 20, 30, 40 µmol/egg) were injected onto the air chamber of 9-day-old (HH 35) chick embryos. After the shell was sealed the injected eggs were incubated for a further 2, 4, 8, 12, 24, 36 or 48 hours. The embryos received a pre-determined 100 µL/egg injection of AAPH while the control group was given the same volume of simple saline (0.72% sodium chloride) only. The experiments were done in triplicates with 20 eggs assigned for each group. The mortality rate was recorded during the experiment, and surviving embryos were harvested for assessment of other parameters.

### Determination of Blood Vessel Density in Chorioallantoic Membrane (CAM) of Chick Embryos

Chick embryos were treated with AAPH (10 µmol/egg at a volume of 100 µL solution) as indicated above, and the surviving embryos were harvested for analysis. Ten embryos in each group were examined. The CAM blood vessels in control group and AAPH treated embryos were photographed by Canon Powershot SX130 IS digital camera (12.1 MPixels) with 12× zoom lens (f3.4–5.6, 28–336 mm) and the areas occupied by the blood vessels were quantified using an image analysis program IPP 5.0 (Image Pro Plus, version 5.0, Media Cybernetics), which automates the assessment of the blood vessel area [25]. The blood vessel density was expressed as the percentage of blood vessel area over the whole area under microscopic field.

### Yolk-sac Blood Vessel Density of Chick Embryos

Eggs containing 3-day-old (HH 18) chick embryos were gently shaken 10 times and then cracked into a petri dish inside a biosafety cabinet. The yolk-sac blood vessels were orientated facing upward when the eggs were cracked. Two silastic rings (inside diameter: 9 mm; outside diameter: 11 mm) were then placed on the vessel region. After 2 h adaptation, different doses of AAPH (4 and 5 µmol) were introduced into one of the two silastic rings, while the same volume of simple saline (control) was added to the other ring. After 12-hour incubation, the embryo reached to HH20. The morphology of the vessel plexus region inside the silastic ring was photographed under a stereomicroscope (Olympus MVX10) with OPTPRO 2007 image acquisition system. The areas occupied by the blood vessel plexus were quantified using an image analysis program IPP 5.0 as indicated above. The blood vessel density was expressed as the percentage of area occupied by blood vessel over the whole area under microscopic field.

### Blood Islands Formation in Chick Embryos

VE-Cadherin is an adhesive molecule that is normally expressed in developing blood islands. Hence, we have performed *in situ* hybridization for VE-Cadherin expression to visualize blood islands formation during vasculogenesis. In early chick (EC) culture [10], half of one side of HH 3^+^ chick embryos was treated with AAPH (5.0 µmol) while the other half was treated with simple saline. To avoid diffusion of AAPH to the control side, a 35 mm plastic dish with a separator in the middle was used for EC culture. After AAPH exposure for 24 hours, the extra-embryonic areas reached stage HH 8, where blood islands were mainly located. Then the embryos were fixed with 4% paraformaldehyde (PFA). Whole-mount *in situ* hybridization of chick embryos (10 embryos for each group) was done as previously described [26]. Digoxigenin-labeled riboprobes were synthesized against VE-Cadherin. The whole-mount stained embryos produced were photographed under a stereomicroscope (Olympus MVX10) and then frozen sections were prepared. The stained embryos were sectioned at a thickness of 15–20 µm on a cryostat microtome (Leica CM1900). These sections were photographed and the areas occupied by blood islands were quantified using the image analysis program IPP 5.0 as indicated above. The density of blood islands that have developed during vasculogenesis was expressed as the percentage of the area occupied by the blood islands over the total area of the microscopic field.

### Morphological Examination of the Embryonic Heart

The effect of AAPH on the embryonic heart was examined. In this experiment, 100 µL of AAPH (1.8 or 2.6 µmol) was injected into the air chamber of fertilized eggs containing HH 35 chick embryos. The injections were repeated on alternative days or consecutive 5 days. Control eggs were injected with the same volume of simple saline. The sample size was 10 for each treatment group. At the end of experimentation, the hearts were harvested from all surviving embryos (about HH 40). These hearts were weighed, photographed and then immediately fixed in 4% PFA. Whole-mount embryonic hearts were photographed using stereoscope fluorescence microscope (Olympus MVX10). They were then dehydrated, embedded in paraffin wax and serially sliced at mid-ventricular level at 5 µm. For histology, the sections of coronal plane were de-waxed in xylene, rehydrated and stained with hematoxylin and eosin (H&E) dye [22]. The sections were photographed using a fluorescent microscope (Olympus IX50) with the NIS-Elements F3.2 software package. All histologic sections are presented with the atria on top and the left ventricle on the left. The thickness of the left and right ventricular walls, and interventricular septum (heart was orientated maximally along its longitudinal axis) were determined using an IPP 5.0 software. Ten different equidistance sites of the ventricular walls and interventricular septum were measured and the average distance was taken as the thickness of ventricular walls or interventricular septum. Myocytes size analysis was further performed under the same microscopic area on the same position of coronal plane sections. Average myocytes surface area was calculated as total area of microscopic field to the total number myocytes.

### Measurement of MDA and ORAC Contents

Oxidative stress was assessed by measuring the MDA content and ORAC levels in CAM and the heart of embryos. Simple saline and AAPH (10 µmol/egg) were administered to 9-day-old (HH 35) chick embryos, with 10 embryos in each group. The embryos were incubated for 2, 4 and 8 hours after saline and AAPH treatment. MDA contents and thicknesses of CAM were determined, while heart homogenate was used in MDA and ORAC assay. The MDA content was determined according to instructions provided in the MDA kit, after protein determination by Coomassie brilliant blue kit. Automated ORAC assay on heart tissue was performed on a Labsystems Fluoroskan Ascent plate reader (Labsystems Co.) with fluorescent filters (Infini-teF200, excitation wavelength, 485 nm; emission wavelength, 527 nm) [27]. Fluorescein was used as a fluorescence probe, and the reaction was initiated following AAPH treatment. Trolox was used as a control. The results of control and AAPH treated samples were analyzed according to differences of their respective fluorescein decay curves.

### Statistical Analysis

The experimental values were given as means ± SD. Statistical difference between two means was evaluated using SPSS13.5 software. Results were only considered significantly different at p<0.05.

## References

[1] Valko M, Leibfritz D, Moncol J, Cronin MTD, Mazur M, et al (2007) Free radicals and antioxidants in normal physiological functions and human disease. Int J Biochem Cell Biol 39: 44–84. 16978905 10.1016/j.biocel.2006.07.001

[2] Abramov JP, Wells PG (2012) Embryoprotective role of endogenous catalase in acatalasemic and human catalase-expressing mouse embryos exposed in culture to developmental and phenytoin-enhanced oxidative stress. Toxicol Sci 120: 428–438. 10.1093/toxsci/kfr007 21252394

[3] Wan J, Winn LM (2006) In utero–initiated cancer: The role of reactive oxygen species. Birth Defects Res C Embryo Today 78: 326–332. 17315246 10.1002/bdrc.20080

[4] Dennery PA (2007) Effects of oxidative stress on embryonic development. Birth Defects Res C Embryo Today 81: 155–162. 17963268 10.1002/bdrc.20098

[5] Covarrubias L, Hernández-García D, Schnabel D, Salas-Vidal E, Castro-Obregón S (2008) Function of reactive oxygen species during animal development: Passive or active? Dev Biol 320: 1–11. 18555213 10.1016/j.ydbio.2008.04.041

[6] Li XD, Qin Y, He RR, Kurihara H (2011) Research progress on oxidative stress models of chicken embryo. Chin Pharmacol Bull 27: 5.

[7] Noguchi N, Yamashita H, Gotoh N, Yamamoto Y, Numano R, et al (1998) 2,2′-Azobis (4-Methoxy-2,4-Dimethylvaleronitrile), a new lipid-soluble azo Iinitiator: application to oxidations of lipids and low-density lipoprotein in solution and in aqueous dispersions. Free Radic Biol Med 24: 259–268. 9433901 10.1016/s0891-5849(97)00230-x

[8] Tang YZ, Liu ZQ (2007) Free-radical-scavenging effect of carbazole derivatives on AAPH-induced hemolysis of human erythrocytes. Bioorg Med Chem 15: 1903–1913. 17236778 10.1016/j.bmc.2007.01.007

[9] Wang YM, Wang HJ, Peng SQ (2009) In ovo exposure of a Fusarium mycotoxin butenolide induces hepatic and renal oxidative damage in chick embryos, and antioxidants provide protections. Toxicol In Vitro 23: 1354–1359. 19573587 10.1016/j.tiv.2009.06.028

[10] Chapman SC, Collignon J, Schoenwolf GC, Lumsden A (2001) Improved method for chick whole-embryo culture using a filter paper carrier. Dev Dyn 220: 284–289. 11241836 10.1002/1097-0177(20010301)220:3<284::AID-DVDY1102>3.0.CO;2-5

[11] Touyz RM, Schiffrin EL (2004) Reactive oxygen species in vascular biology: implications in hypertension. Histochem Cell Biol 122: 339–352. 15338229 10.1007/s00418-004-0696-7

[12] Weseler AR, Bast A (2010) Oxidative stress and vascular function: implications for pharmacologic treatments. Curr Hypertens Rep 12: 154–161. 20424954 10.1007/s11906-010-0103-9 PMC2876260

[13] Sarre A, Pedretti S, Gardier S, Raddatz E (2010) Specific inhibition of HCN channels slows rhythm differently in atria, ventricle and outflow tract and stabilizes conduction in the anoxic-reoxygenated embryonic heart model. Pharmacol Res 61: 85–91. 19818405 10.1016/j.phrs.2009.09.007

[14] Torry DS, Hinrichs M, Torry RJ (2004) Determinants of Placental Vascularity. Am J Reprod Immunol 51: 257–268. 15212678 10.1111/j.1600-0897.2004.00154.x

[15] Yamamoto Y, Niki E, Eguchi J, Kamiya Y, Shimasaki H (1985) Oxidation of biological membranes and its inhibition. Free radical chain oxidation of erythrocyte ghost membranes by oxygen. Biochim Biophys Acta 819: 29–36. 3840035 10.1016/0005-2736(85)90192-0

[16] Hiramoto K, Johkoh H, Sako KI, Kikugawa K (1993) DNA breaking activity of the carbon-centered radical generated from 2, 2′-azobis (2-amidinopropane) hydrochloride (AAPH). Free Radic Res Commun 19: 323–332. 8314113 10.3109/10715769309056521

[17] Landi L, Fiorentini D, Galli MC, Segura-Aguilar J, Beyer RE (1997) DT-Diaphorase maintains the reduced state of ubiquinones in lipid vesicles thereby promoting their antioxidant function. Free Radic Biol Med 22: 329–335. 8958158 10.1016/s0891-5849(96)00294-8

[18] Kuo CT, Morrisey EE, Anandappa R, Sigrist K, Lu MM, et al (1997) GATA4 transcription factor is required for ventral morphogenesis and heart tube formation. Genes Dev 11: 1048–1060. 9136932 10.1101/gad.11.8.1048

[19] Bae YS, Oh H, Rhee SG, Yoo YD (2011) Regulation of reactive oxygen species generation in cell signaling. Mol Cells 32: 491–509. 22207195 10.1007/s10059-011-0276-3 PMC3887685

[20] Gavaller H, Sepp R, Csanady M, Forster T, Nemes A (2011) Hypertrophic cardiomyopathy is associated with abnormal echocardiographic aortic elastic properties and arteriograph-derived pulse-wave velocity. Echocardiography 28: 848–852. 21827547 10.1111/j.1540-8175.2011.01469.x

[21] Abe C, Uto Y, Nakata E, Hori H (2009) Development of an in vivo evaluation system of antioxidants for their vascular protective activities using the chick embryonic chorioallantoic membrane. Bull Inst Technol Sci Univ Tokushima 54: 70–74.

[22] Ma ZL, Qin Y, Wang G, Li XD, He RR, et al (2012) Exploring the caffeine-induced teratogenicity on neurodevelopment using early chick embryo. Plos one 7: e34278. 22470550 10.1371/journal.pone.0034278 PMC3314624

[23] Hamburger V HH (1951) A series of normal stages in the development of the chick embryo. J Morphol 88: 49–92. 24539719

[24] Li XD, He RR, Qin Y, Tsoi B, Li YF, et al (2012) Caffeine interferes embryonic development through over-stimulating serotonergic system in chicken embryo. Food Chem Toxicol 50: 1848–1853. 22449533 10.1016/j.fct.2012.03.037

[25] Doukas CN, Maglogiannis I, Chatziioannou A, Papapetropoulos A (2006) Automated angiogenesis quantification through advanced image processing techniques. Conf Proc IEEE Eng Med Biol Soc 1: 2345–2348. 17946107 10.1109/IEMBS.2006.260675

[26] Henrique D, Adam J, Myat A, Chitnis A, Lewis J, et al (1995) Expression of a Delta homologue in prospective neurons in the chick. Nature 375: 787–790. 7596411 10.1038/375787a0

[27] He RR, Yao XS, Li HY, Dai Y, Duan YH, et al (2009) The anti-stress effects of Sarcandra glabra extract on restraint-evoked immunocompromise. Biol Pharm Bull 32: 247–252. 19182384 10.1248/bpb.32.247

